# Meta-All: a system for managing metabolic pathway information

**DOI:** 10.1186/1471-2105-7-465

**Published:** 2006-10-23

**Authors:** Stephan Weise, Ivo Grosse, Christian Klukas, Dirk Koschützki, Uwe Scholz, Falk Schreiber, Björn H Junker

**Affiliations:** 1 Leibniz Institute of Plant Genetics and Crop Plant Research, Corrensstr. 3, 06466 Gatersleben, Germany; 2 Brookhaven National Laboratory, 50 Bell Avenue, Upton, NY 11973, USA

## Abstract

**Background:**

Many attempts are being made to understand biological subjects at a systems level. A major resource for these approaches are biological databases, storing manifold information about DNA, RNA and protein sequences including their functional and structural motifs, molecular markers, mRNA expression levels, metabolite concentrations, protein-protein interactions, phenotypic traits or taxonomic relationships. The use of these databases is often hampered by the fact that they are designed for special application areas and thus lack universality. Databases on metabolic pathways, which provide an increasingly important foundation for many analyses of biochemical processes at a systems level, are no exception from the rule. Data stored in central databases such as KEGG, BRENDA or SABIO-RK is often limited to read-only access. If experimentalists want to store their own data, possibly still under investigation, there are two possibilities. They can either develop their own information system for managing that own data, which is very time-consuming and costly, or they can try to store their data in existing systems, which is often restricted. Hence, an out-of-the-box information system for managing metabolic pathway data is needed.

**Results:**

We have designed META-ALL, an information system that allows the management of metabolic pathways, including reaction kinetics, detailed locations, environmental factors and taxonomic information. Data can be stored together with quality tags and in different parallel versions. META-ALL uses Oracle DBMS and Oracle Application Express. We provide the META-ALL information system for download and use. In this paper, we describe the database structure and give information about the tools for submitting and accessing the data. As a first application of META-ALL, we show how the information contained in a detailed kinetic model can be stored and accessed.

**Conclusion:**

META-ALL is a system for managing information about metabolic pathways. It facilitates the handling of pathway-related data and is designed to help biochemists and molecular biologists in their daily research. It is available on the Web at  and can be downloaded free of charge and installed locally.

## Background

Modern molecular biological research produces large amounts of data. One main problem remains the elucidation and representation of relationships between the compounds of biological systems. A large number of information systems holding data from molecular biology has been developed by public and private research projects [1]. A rapidly growing field is the collection of data associated to metabolic pathways, available from information systems such as KEGG [2], BRENDA [3], UM-BBD [4] or Reactome [5]. Depending on the requirements defined upon the initiation of these projects, the features and implementations differ significantly among these systems. For example, BRENDA holds detailed kinetic information about enzymes, whereas KEGG contains maps of metabolic pathways and detailed descriptions about their elements. Often, the existing systems are not well structured, because they have been grown over the years. Hence, they are not able to store all necessary information [6]. For example, many of the systems are not capable of describing the complexity of higher organisms. Especially the differentiation between loci inside of organisms, the consideration of developmental stages and stress factors, and detailed representation of kinetics and regulation is only partially possible. Additionally, there are errors resulting from faulty text-mining procedures or genome-based pathway predictions, which make the data only conditionally useful. Hence, existing systems often represent collections of reference pathways. This is useful for getting an idea of the metabolic processes as in the case of the well-known Boehringer charts [7,8], however, in many cases it is insufficient. Only rudimentary details are stored about the real location of these processes, such as the organism, tissue, cell type and compartment, while comprising comprehensive information about pathway structures in general. Also, only sparse information is given about circumstances under which the data points were determined, such as growth conditions or sampling time. Hence, some of the represented pathways are non-occurring in reality. As an exception the SABIO-RK system [9] should be mentioned, which focuses on the storage of fine-grained and high-quality data in a central instance.

Another important problem biochemists and molecular biologists are faced with is the management of their own data. Currently, experimentalists can develop an own information system for managing that own data, but this is very time-consuming and costly. An alternative would be to try to get their data into existing systems. Often, this is not possible. In case that scientists have different opinions about certain items, pathway data needs to be stored in different parallel versions. However, this is not possible with most of the existing systems.

In an attempt to overcome these problems and to provide the scientific community with a software system that meets their requirements, we initiated the META-ALL project. META-ALL is designed for management of detailed information about metabolic pathways, including reactions, translocations, substances, pathways, locations and kinetic parameters. The system contains a versioning system and a Web interface for entering and querying of data.

Meta-All is a software package that is initially distributed with an example data-set only. It can be installed locally to help biologists and biochemists in their daily research. The user can enter own experimental data as well as data sets from other sources such as publications or databases. Once the user's instance of Meta-All is populated with sufficient amounts of data, a variety of complex queries will be possible due to the detailed structure of the database schema. Examples for such queries are dependent on the filling of the system, and could be: "What is the *K*_*m *_value for enzyme *x *in compartment *y *of organ *z *of organism *w*?" or "If a value for organism *x *is missing, what value was measured in the closest relative (in taxonomical sense) of that organism?"

In this paper, at first we give an overview about the technical background of the system. In the second part we describe the database schema, before we provide details about the user-interface we developed for managing the contents of the database. Finally, we discuss our system in relation to other systems.

## Implementation

### Technical design of Meta-All

META-ALL uses the Oracle database management system, which is extensively used in academia and industry. Furthermore, Oracle provides several useful tools for easy creation of user interfaces and a security concept for protecting selected data. For the user-frontend, we use Oracle Application Express [10]. It allows the wizard-aided creation of Web-interfaces based on the underlying database schema with the help of a set of components, such as graphical and non-graphical reports, forms and trees, which can be easily integrated into different areas (regions) of Web pages. We use the user management provided by Oracle Application Express for allowing the creation of users with different rights such as read/write or read-only access. Oracle Application Express addresses also the concerns of multiple-user access with built-in check constraints. Hence, it is possible for multiple users to use META-ALL at the same time; it is ensured that two users cannot edit the same data simultaneously and that one user cannot overwrite the respective changes of the other user.

META-ALL can be accessed in two ways. First, we provide a demo instance of META-ALL on our server which is accessible via our project Web page [11]. This instance is intended to provide an overview about the abilities of META-ALL. A guest user account with the username "*guest*" and the password "*meta-all*" allows the user to see a pathway from sucrose breakdown in potato as a test data set [12]. The demo instance will be reset at regular intervals. The second possibility is to download META-ALL and to install it locally. That requires Oracle DBMS and Oracle Application Express running. This can be either a commercial version of Oracle software or an Oracle Express Edition [13], which is a freely available lightweight Oracle DBMS coming along with an integrated Application Express installation. The META-ALL application including the initial data set and the installation procedure is available for download free of charge at our project Web page.

### Database schema

**Figure 1:**
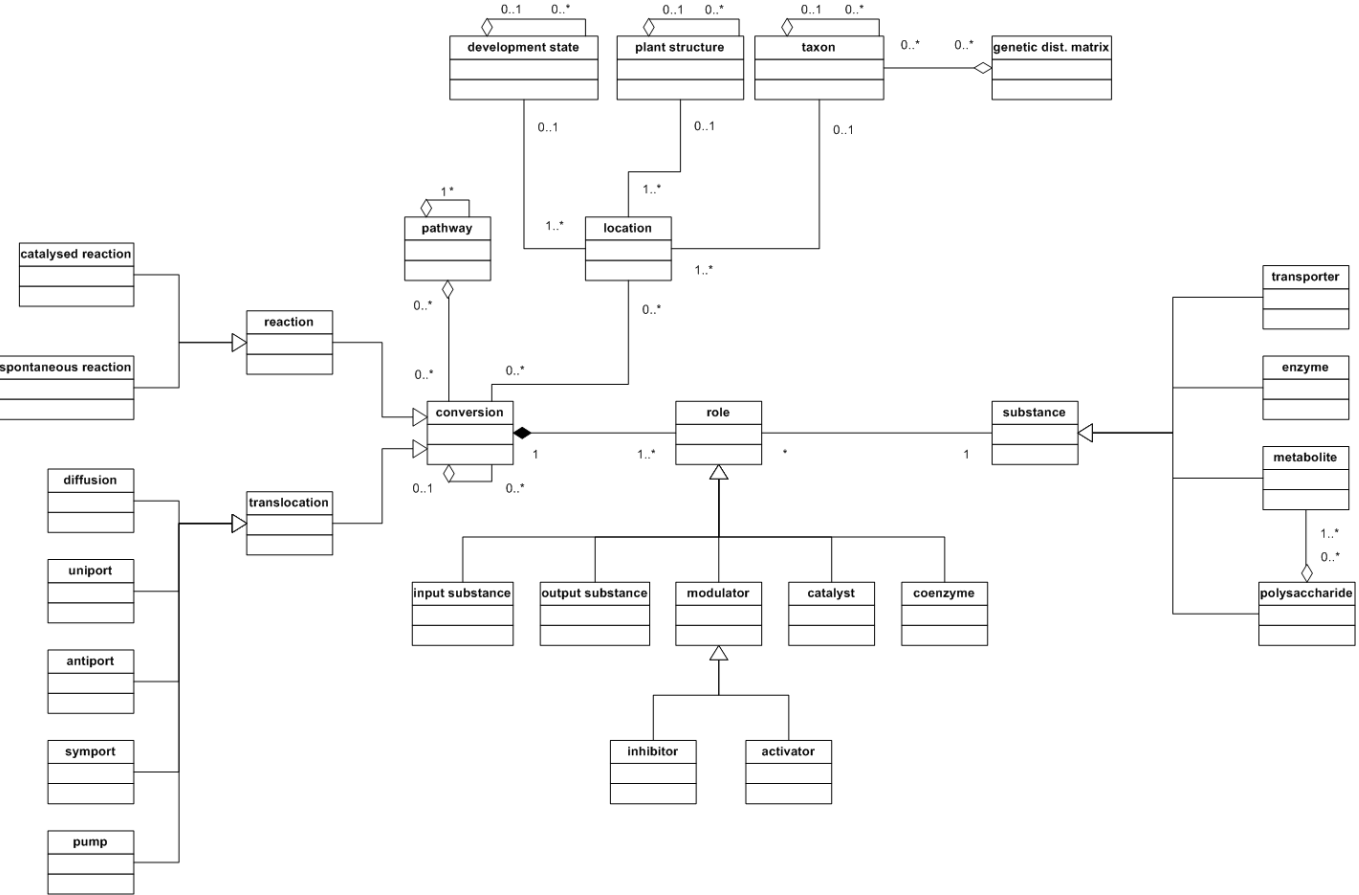
**Simplified schema of the Meta-All database**. Simplified UML [30] structure diagram of the database schema. The complete schema is available at the META-ALL project Web page. Rectangles symbolise classes (e.g. substance) without attributes and methods. Lines between classes symbolise relationships between the classes, called associations. Small diamonds at one of the ends of a line symbolise part-of relations. A black diamond means a composition (the single parts cannot exist on their own), an open diamond means an aggregation (the single part can exist on its own). A line with a triangle at one of the ends symbolises an inheritance relation (e.g. both metabolite class and enzyme class belong to the substance class). Numbers next to lines specify the allowed cardinalities for an instance of that class, for example, the 1..* at the line between metabolite and macromolecule means that a macromolecule consists of at least one (1) metabolite up to an unlimited (*) number of metabolites.

The database schema is divided into several parts. The main parts are conversions, substances, pathways, locations (taxonomy, developmental stage, cytology), references and versioning. A simplified version reflecting all objects storable in the database schema is shown in Fig. 1. The complete relational schema comprising 51 database tables is available at the META-ALL project Web page. To develop the database schema of META-ALL, we compared several existing information systems storing data about metabolic pathways and extensively discussed with experimentalists about their needs and wishes. The schema was improved in multiple cycles, according to the spiral model [14] popular in software engineering.

#### Conversions and substances

The central parts of the schema are *conversions *and *substances*. A conversion is a reaction or a translocation, both of them either actively (enzyme-catalysed or transporter-mediated, respectively) or passively (spontaneous or by diffusion, respectively). All necessary information can be stored with every conversion: name and synonym(s), formula, type (reaction, translocation) and subtype (kinetic reaction type for enzymes, type of transporter). Conversions are categorised into pathways and pathways into super-pathways. Furthermore, some specific information can be included, e.g. for the layout of cycles (if there is a clockwise or an anti-clockwise cycle, or if it is an open or closed one). The substance is an umbrella term for transporters, enzymes, metabolites and macromolecules, the latter of which consists of one or more types of metabolite units. Each substance plays a certain role in a conversion, which can be catalyst in case the substance is an enzyme, or substrate (= input substance) in case the substance is a metabolite. Furthermore, a metabolite can be a modulator (activator or inhibitor) of a reaction, the product (= output substance) or act as a coenzyme. Each of the role information can be enriched by detailed information such as *V*_*max *_values, affinity constants, etc. Each piece of information is assigned to the location information and publications.

#### Locations and taxonomy

Conversions take place at different locations inside an organism depending on the developmental state and environmental effects, and the database schema reflects as many of these parameters as possible. We focused on the use of existing ontologies in order to have a controlled vocabulary allowing the comparison of data from different sources. For our instance of META-ALL we decided to use PlantOntology [15] terms to distinguish cytological aspects of plants and also for developmental stages, because the scientific focus of our institute is plant research. The META-ALL system can be easily extended by other ontologies or user-defined terms, respectively. To determine the taxonomy of the organisms, we use the NCBI taxonomy ID [16] enriched by established attributes such as family, genus or species. Additionally, META-ALL allows the storage of genetic distance matrices of different organisms. The user can infer these matrices for example by comparison of gene sequences from the organisms stored in the database. Thus, in case a pathway is poorly investigated in one organism, this feature allows the recruitment of data from a phylogenetically closely related species.

#### Reference information

As publications represent probably the most important knowledge source, it is crucial to store reference information with as many data points as possible. We decided to use the widely accepted standard of PubMed IDs instead of developing an integrated publication management. Hence, META-ALL stores the PubMed ID for each publication and a text field is provided to store necessary information for yet unpublished data. The record has to be updated manually once a PubMed ID is available. If using the META-ALL Web-Interface described below, the user is shown the PubMed ID and, if existing, the remark field. The NCBI Web page with the abstract of the publication can be opened in a new window using a provided link.

#### Quality tags and data versioning

The data for which META-ALL is intended usually has different quality levels. Hence, the storage of additional quality information and the possibility to store curated data, as it was pointed out in [17], is required. In addition, there is a strong need for a versioning system for a pathway database like META-ALL, because biochemical data is sometimes ambiguous and even long-established views are subject to change. We need to be able to store different revisions of pathways as scientists tend to have different opinions about certain issues. There are several possibilities for data versioning [18,19]. Linear versioning could be used, which means that each version has only one successor. In contrast, as one of our requirements to the system was to enable parallel instances of pathways, a hierarchical versioning would be a benefit. Both linear and hierarchical versioning can be subdivided into continuous versioning and discrete versioning. Continuous versioning means that every simple change is a new version. In contrast, if using discrete versioning, which was used for META-ALL, a new version is created at a certain interval. In the context of META-ALL, the decision to create a new version is made by the user. All versioned values are stored in the same table distinguished by the status. We do not work with shadow tables archiving older values, because then only one value would actually be available. The term "status" as used in META-ALL is a label comprising the time-stamp at which a new version was created. In addition, it contains information such as the author of the version, the originality of the data (e.g. in-house data, public database), the quality of the data (e.g. hand-curated, imported from external source, putative) and the ID of the status record the newly generated status (or version) is based on. The labelled data sets can be improved gradually. Additionally, old pathways can be kept for publication purposes (in case the pathway has been improved in the meanwhile).

### The Meta-All Web-Interface

**Figure 2:**
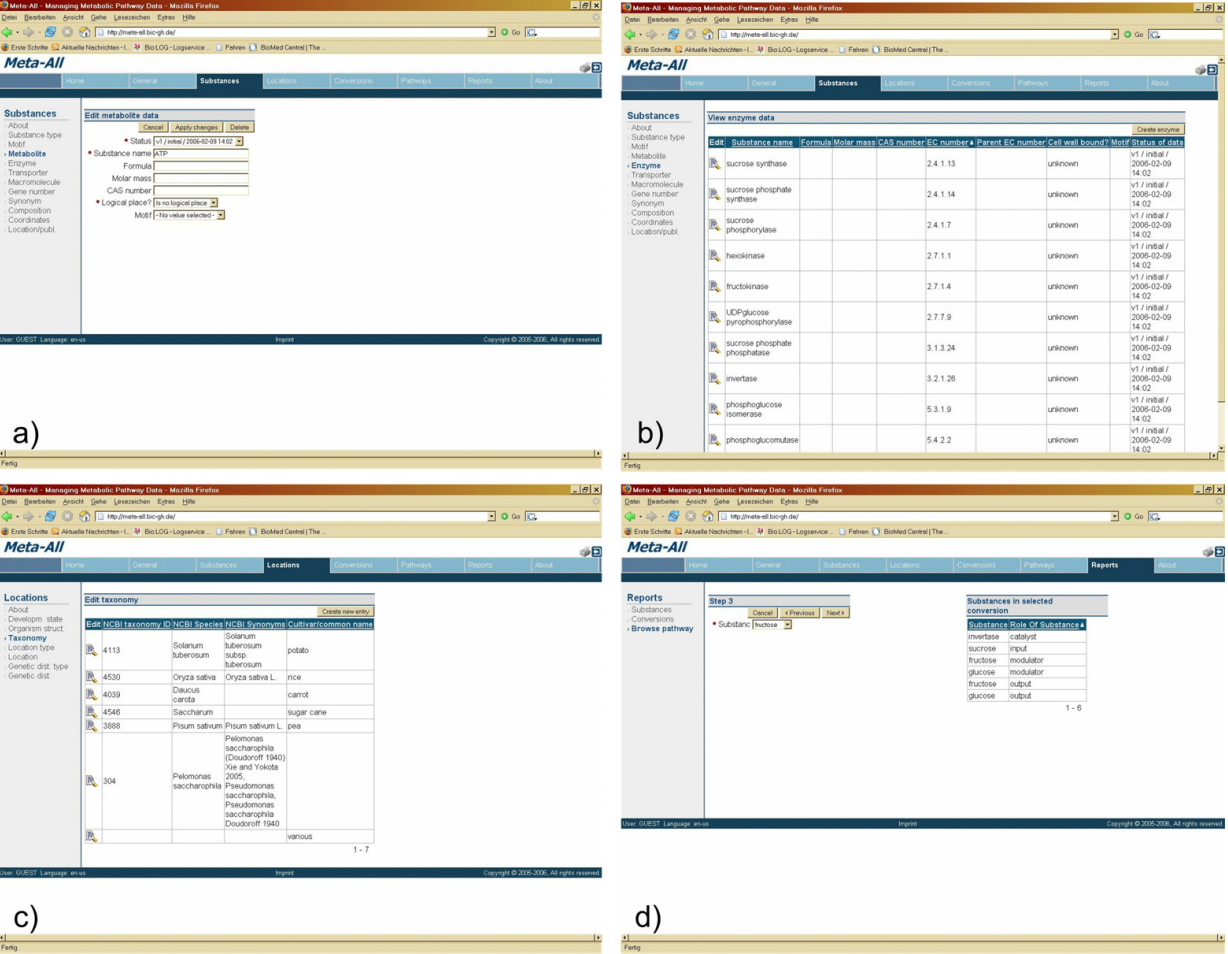
**Screenshot of the Meta-All Web-Interface**. The Web-Interface is divided into several parts, which correspond with the main parts of the database schema as described in Fig. 1. There are tabs for substances, locations, conversions, pathways and the management of versions and publications. These tabs open sub-pages each with several items belonging to the particular part of the schema. **a) **shows an input form for inserting data about metabolites, and **b) **is a simple report listing enzymes. **c) **is showing taxonomy information of the organisms stored in the demo instance of META-ALL, and **d) **shows a step from a wizard for browsing top-down through a pathway. On the left side, there is a select list for choosing one of the substances listed in the report on the right side. These substances belong to a conversion selected in the previous step. By clicking on the "next"-button, details of the chosen substance will be shown.

**Figure 3:**
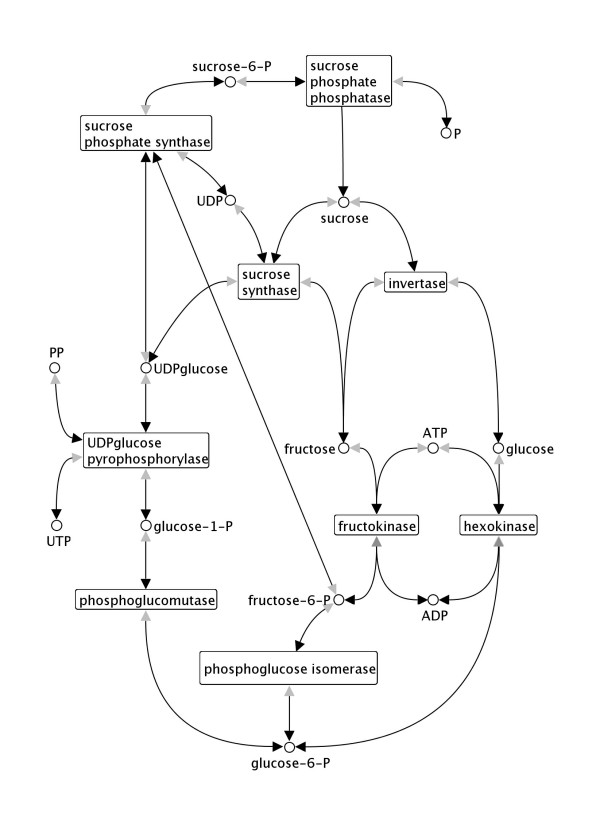
**Data from Meta-All visualised in Vanted**. Visualisation of the sucrose breakdown pathway in the potato tuber [12]. The pathway data stored in META-ALL was exported into an SBML file using the SBML export filter. The file was subsequently loaded into the network visualisation system VANTED [22]. As the SBML format does not specify a layout of the model, an automatic force directed layout was applied which served as a starting point for the manual refinement of the overall model layout.

The META-ALL Web interface is the central user-interface to the META-ALL database (Fig. 2). We used the Application Express [10] technology from Oracle as described above (subsection Technical design of the system). META-ALL comes along with two levels of navigation tabs reflecting the certain parts of the database schema. Each part (level one) consists of several pages (level two). Several forms allow inserting new data, creating and assigning of versions, publications, etc. For example, the user can insert new substances (e.g. metabolites or enzymes). Consecutively, these substances can be assigned with conversion processes, which can be assigned with pathways, super-pathways and so on. All data can be visualised using reports, and selective data can also be edited depending on the authorisation of the user. Several reports are possible inside of META-ALL, e.g. graphical reports about contents of the database as there are enzymes and metabolites, or groupings of reactions regarding to the pathways they belong to. Whole pathways can be selected and then exported from the META-ALL Web-Interface in SBML format [20]. META-ALL comes with the general rate law for a number of standard kinetic types (e.g. Ping-Pong bi bi) using MathML [21]. The user associates a conversion with a kinetic type. When an SBML file is generated for a pathway, META-ALL uses the necessary information such as the binding order of the substrates, which are stored together with the conversion, and fills the according values into the rate law. The SBML file can be loaded into a visualisation system, e. g. VANTED [22], as shown in Fig. 3, or into a simulation system, e. g. COPASI [23].

## Results

META-ALL is an information system for managing data about metabolic pathways, which is available for download and local installation, in order to help experimentalists in their daily research. We provide both the database schema (together with an initial set of data from sucrose breakdown in the potato tuber) and the user interface. It is available from the META-ALL Web page. Additionally, we provide a demo instance of the META-ALL system on our server.

### Case study

The pathway by which sucrose is transformed to starch in developing potato tubers has been subject of numerous studies over several decades (for a review see [24]). A detailed kinetic model has been created [12] in an attempt to better understand the dynamics of this pathway. The model consists of 14 reactions with their according rate-laws, which are further defined by a total of 74 constants. The data contained in the model represents a useful test data set for META-ALL and was thus entered into the system. It is possible to represent all data from the model in META-ALL, including:

• The enzyme name, EC number and stoichiometric formula.

• The kinetic type of the reaction, together with the organism this was determined in.

• The name of each metabolite, the role it plays in which reaction, and the binding- and dissociation order in these reactions.

• The kinetic parameters of each reaction such as binding or inhibitory constants, maximal velocity and equilibrium constant; their value and unit; the organism, tissue, cell, and compartment the constant was measured in.

• For all data, the reference and the according PubMed ID (if available) is given, together with a tag if this kinetic type was measured or estimated.

The structure of the metabolic network has been exported from META-ALL as an SBML file and imported into the data visualisation system VANTED for a visualisation of the pathway. Figure 3 shows the result of the visualisation. While this procedure shows the potential of the system, it should be pointed out that the connection of META-ALL to VANTED is only one of numerous possibilities that could be implemented depending on the preferences of the user.

## Discussion

Although there exist several information systems for storing metabolic pathway data, we decided to develop a new one, because many of the existing systems are not capable of meeting the requirements of experimentalists working on higher organisms. We analysed a variety of existing information systems concerning their advantages and disadvantages and made these information the fundament of our requirement analysis. An exciting study in this field is published in [25].

An information system of high interest is SABIO-RK [9]. It focuses on the curation of data about biochemical reactions, including their kinetic properties. The limitations of SABIO-RK are that data is stored in a central instance with a read-only access and that there is no user interface for inserting data. A system with similar goals as Meta-All is aMAZE [26]. It consists of a WorkBench for storing pathway data and a front-end (LightBench) for accessing this data. Additional components, namely the Snow Workbench and a direct SQL access to the data, are available. To the best of our knowledge, aMAZE cannot store information about reaction kinetics, and is not able to write SBML files. In the current version the aMAZE LightBench can be used for querying data in the central installation in Belgium only. The intension of META-ALL is to support experimentalists in their daily work. While most of the existing systems focus on storing and curating data in a central instance, e. g. KEGG, and can just be accessed read-only, META-ALL can be installed locally, and data can be inserted and edited. Research groups or even whole institutes can manage their "fresh" data, or even data subject to current investigations. The management is supported by versioning and quality tagging. Furthermore, not only reaction data, but also translocation data can be managed with META-ALL.

META-ALL uses an Oracle database management system for persistently storing metabolic pathway data and an Oracle Application Express user interface. An installation instruction containing database schema, user interface and initial data set can be obtained from the META-ALL project Web page. It can be used with either a commercial Oracle license or the Oracle Database 10 g Express Edition, which is available free of charge from Oracle Corporation.

We prepared several reports for the META-ALL user interface and used our system in conjunction with VANTED for pathway visualisation. We plan to connect META-ALL to the Systems Biology Modelling Environment (SYBME) [27] to further support the user in the kinetic metabolic modelling. Within SYBME, a user will be able to browse through the information of metabolites and reactions available in his/her META-ALL instance, may combine this information into a kinetic model and can finally visualise and simulate the model with the two connected simulators, GEPASI [28] and JARNAC [29].

For the future, we plan to facilitate the following application using META-ALL: the semi-automatic generation of kinetic models of a certain pathway. The procedure for this task will be as follows: the user enters the pathway that should be modelled and defines the boundaries of the model (substrates, products). An application of META-ALL then queries the database for all information about the enzymes present in this pathway at a specific location in a given organism. If a certain kinetic parameter is not available for an enzyme, it will be possible to take the data from the taxonomically nearest neighbour species for which the data is available with the help of the genetic distance matrix included in META-ALL. This is a common approach for constructing kinetic models, which is normally done through tedious literature searches. The data can then be presented to the user who can decide which data to keep and which to change. Subsequently, the model could be exported in standard formats such as SBML. We are aware that this is a future application implying that the database is filled with a sufficient amount of data, which is in the responsibility of the user. The user could, for example, import large datasets from existing sources and improve the data step by step. This procedure is supported by the quality and versioning tagging described in subsection Database schema.

For the daily work, the existing Web-Interface should be appropriate. In the future, we plan to expand META-ALL by an SBML importer allowing the import of larger amounts of data at once.

## Conclusion

In this paper, we presented META-ALL, a metabolic pathway information system, which can be downloaded and installed locally. It is intended to support biochemists and molecular biologists in their daily research by providing a platform for the management of detailed information about metabolic pathways, including reactions, translocations, substances, pathways, locations and kinetic parameters. META-ALL contains a versioning system, quality tags and a Web interface for entering and querying of data. Pathways can be exported into SBML files for use in visualisation or simulation tools.

## Availability and requirements

• **Project name: **META-ALL

• **Project home page: **

• **Operating system(s): **Client: only a Web browser required; Server: OS depending on the Oracle installation, e. g. Microsoft Windows, Linux

• **Programming language: **User-interface using the Oracle Application Express technology and SQL, PL/SQL

• **Other requirements: **Oracle DBMS 9i/10g (version 9.2.0.3 and higher) and Oracle Application Express 2.0 license if available, or Oracle Database 10g Express Edition (free of charge entry-level Oracle DBMS, including Application Express)

• **License: **Apache License, Version 2.0

• **Any restrictions to use by non-academics: **According to license

## Authors' contributions

All authors participated in the design of the system. SW implemented the system. SW and BHJ designed the database schema and drafted the manuscript. All authors read and approved the final version.
